# Repair of primary and incisional hernias using composite mesh fixed with absorbable tackers: preliminary experience of a laparoscopic approach with a newly designed mesh in 29 cases

**DOI:** 10.1007/s13304-017-0444-x

**Published:** 2017-04-13

**Authors:** Ferdinando Agresta, Alice Marzetti, Silvia Vigna, Daniela Prando, Raffaele Porfidia, Salomone Di Saverio

**Affiliations:** 1 Department of General Surgery, ULSS19 del Veneto, Adria, RO Italy; 2 Department of General Surgery, Emergency and Trauma Surgery Unit, C. A. Pizzardi Maggiore Hospital Trauma Center, AUSL Bologna, 40100 Bologna, Italy

**Keywords:** Laparoscopy, Abdominal hernias, Absorbable tackers

## Abstract

Outcome of primary and incisional hernia repair is still affected by clinical complications in terms of recurrences, pain and discomfort. Factors like surgical approach, prosthesis characteristics and method of fixation might influence the outcome. We evaluated in a prospective observational study a cohort population which underwent primary and incisional laparoscopic hernia repair, with the use of a composite mesh in polypropylene fixed with absorbable devices. We focused on assessing the feasibility and safety of these procedures; they were always performed by an experienced laparoscopic surgeon, analyzing data from our patients through the EuraHS registry. Seventy nine procedures of primary and incisional hernia repair were performed from July 2013 to November 2015 at Santa Maria Regina degli Angeli Hospital in Adria (RO). All cases have been registered at the EuraHS registry (http://www.eurahs.eu); among them, we analyzed 29 procedures performed using a new composite polypropylene mesh (CMC, Clear Composite Mesh, DIPROMED srl San Mauro Torinese, Turin, Italy), fixed with absorbable tackers (ETHICON, Ethicon LLC Guaynabo, Puerto Rico 00969). We performed 23 incisional hernia repairs, 4 primary hernia repairs (1 umbilical, 2 epigastric and 1 lumbar hernia) and 2 parastomal hernia repairs. The median operation time was 65.1 min for elective and 81.4 min for urgent procedures (three cases). We had two post-operative complications (6.89%), one case of bleeding and another case of prolonged ileus successfully treated with conservative management. We had no recurrences at follow-up. According to QoL, at 12 months patients do not complain about any pain or discomfort for esthetic result. Laparoscopic treatment of primary and incisional hernia with the use of composite mesh in polypropylene fixed with absorbable devices is feasible and safe.

## Introduction

Outcome of primary and incisional hernia repair is still affected by significant incidence of clinical complications in terms of recurrences, pain and discomfort. Factors like surgical technique and/or prosthesis characteristics and method for fixation might influence surgical outcome.

It is well known that the best alternative for quality control of medical devices is to collect data from a comprehensive registry; so far, starting in September 2013 all our patients operated for abdominal hernias, both incisional as primary, are registered within the EuraHS registry [1]. This register is an international online platform for recording and outcomes measurement of hernia operations, which includes a set of definitions and classifications for use in clinical research on abdominal wall hernias in adult patients older than 18 years. According to this register, the outcome of operations will depend on the interaction between the three entities involved that are patient–procedure–prothesis and their different variables that all might have influence on the outcome. This register requires completion of a follow-up at 6 weeks and 12 and 24 months, using “EuraHS-QoL” score for quality of life before and after surgery. In the literature, several generic quality of life (QoL) scores, e.g., the Sf-36 score have been used after surgery. However, for QoL evaluation after hernia repair not all scores have been proven useful. Therefore, the EuraHS working group proposes a new and open EuraHS-QoL score, specifically targeting patients that underwent abdominal wall hernia repair pre- and post-operatively. The EuraHS-QoL score is based on a Numerical Rating Scale for three dimensions: pain at the site of the hernia or the hernia repair (defined as pain at rest plus pain during activities and pain felt during the last week); restriction of activities (defined as restriction from daily activities inside the house; restriction outside the house such as walking, biking, driving; Restriction during sports if performed and restriction during heavy labor if performed); cosmetic discomfort (defined as shape of the abdomen and site of the hernia). The validation of the EuraHS-QoL score will be part of the research conducted by the EuraHS working group. We focused on the feasibility, safety and potential benefits of a laparoscopic repair of abdominal wall hernias, both incisional and primary, using a composite polypropylene mesh fixed with absorbable tacks analyzing our database through EuraHS registry [1]. The secondary end point of the study is the evaluation of the quality of life according to the three dimensions above reported.

## Materials and methods

Between July 2013 and November 2015, a total of 79 procedures of primary and incisional hernia repairs were performed at Santa Maria Regina degli Angeli Hospital in Adria (RO); All these cases have been registered at the EuraHS register (www.eurahas.eu). We aimed to analyze 29 out of these procedures that were performed laparoscopically using a new composite polypropylene mesh (CMC, Clear Composite Mesh, DIPROMED srl San Mauro Torinese, Turin, Italy), fixed with absorbable tackers (ETHICON SECURESTRAP, Ethicon LLC Guaynabo, Puerto Rico 00969).

If no general or local (no secure area where establishing a pneumoperitoneum) contraindications, laparoscopy is our preferred approach to repair abdominal wall hernias. A previous mesh repair, also intra-abdominal one, location and defect size are not considered contraindication for a laparoscopic repair too.

All patients were assessed by standard pre-operative work-up for laparoscopic procedures (chest X-ray, ECG and routine blood tests).

The patients have been regularly followed up including quality of life (EuraHS-QoL Score), regarding the CMC cases with follow up completed at 6 weeks and 12 months.

## Technique

In laparoscopic abdominal hernia repair, the patient is supine with the arms alongside the body. A nasogastric tube and urinary catheter are inserted after induction of anesthesia (in case of suprapubic incisional hernia) and removed at the end of the procedure. The procedure is performed through 3–4 abdominal trocars (one of 15 mm and two to three of 5 mm), and all the instruments and optic (30) are of 5 mm. Adhesiolysis is performed if necessary using diathermy to have all the scar’s surface free. We do not usually dissect the hernia sac. The mesh is introduced into the abdominal cavity through the 15-mm trocar. The mesh is then placed over the defect with at least 5 cm of overlap at all sides. We temporarily suspend the mesh to the abdominal wall using three sutures passed transcutaneously on the cranial edge, caudal edge as well as in the central part of the mesh which we remove at the end of the surgery. During this step we use to lower the intra-abdominal pressure to 10 mmHg. These maneuvers allow us to obtain a perfect calibration of the mesh on the hernia and to easily fix it with tacks.

Fixation of the mesh is achieved by 5-mm absorbable tackers. A concentric ring of tackers is placed in the peripheral margin of the mesh. Hemostasis is achieved before removal of the trocars.

## Results

Sex distribution of the patients showed 14 men and 15 women with a median BMI of 28.5 (range 24.3–32.4). We performed 23 incisional hernia repairs, 4 primary hernia repairs (one umbilical, one lumbar and two epigastric hernias) and 2 parastomal hernia repairs (Table 1). All procedures were under general anesthesia. The median operation time was 65.3 (range 45.2–72.5) minutes for elective (n.26, 89.65%) and 81.2 (range 59.5–90.3) minutes for urgent procedures (n.3, 10.34%) The median length of stay was 3.07 days (range 3–5.5) in election and 6.88 days in urgent setting (range 6–9.5).

**Table 1:** Type, localization and size of hernia according to the European Hernia Society

	Incisional hernia	Primary hernia	Parastomal hernia
LOCALIZATION	n.2 L1 n.2 L2R n.2 L3 n.1 L3R n.1 L4R n.1 M1 n.2 M2 n.3 M2M3 n.2 M3 n.2 M4 n.2 M3M4 n.1 M5 n.2 M4M5	n.3 M n.1 L	2 PHIII-
WIDTH/LENGTH OF HERNIA (cm)(range)	10.6 (4–20)/18.7 (4–30)	9.2 (8–10)/12.3 (10–14)	5/10
MEDIAN SIZE (cm^2^)	104	108	50

*M* midline (1: subxyphoidal, 2: epigastric, 3: umbilical, 4: infraumbilical, 5: suprapubic), *L* lateral/lumbar (1: subcostal, 2: flank, 3: iliac), *PH* primary parastomal hernia size ≥5 cm

As for comorbidities, three patients (10.34%) had cardiac disease, two patients (6.89%) had arterial hypertension and one patient (3.44%) had pulmonary disease.

Concomitant surgery was performed in six cases (20.68%): three concomitant procedures of cholecystectomy, two procedures of inguinal hernia repair, one urological procedure.

We had two post-operative complications (6.89%): one case of bleeding and another case of post-operative prolonged ileus both successfully treated conservatively (Clavien-Dindo class 1). No mesh-related complications (prosthesis rejection and/or infection) occurred. None of the patients developed a seroma at 6-week follow-up (Table 2).

**Table 2:** Main patients’ characteristics and main results

Patients	29
Male/female	14/15
Median hospital stay elective/emergency (range)	3.07 (3–5.5)/6.88 (6–9.5) days
BMI	28.5 (range 24.3–32.4)
Comorbidities	6 patients (20.6%)
Emergency	3 (10.34%)
Median operative time elective/emergency (range)	65.3 (45.2–72.5)/81.2 (59.5–90.3) minutes
Concomitant surgery	6 procedures (20.68%)
Post-op complications	2 (6.89%)
Recurrences (at 12-month follow-up)	0

There was no conversion to open repairs or deaths in our series.

All patients have been followed up for 12 months. According to EuraHS-QoL Score, in the follow-up at 6 weeks, pain in rest is about 0.8 and raises to 1.50 during physical activities with a restriction of activities score of 0.4 and a score for cosmetic discomfort of 1; at the 12-month follow-up the score for pain in rest is 0.04 and 0.01 during activities, with a restriction of activities score of 0.1 and the cosmetic discomfort one of 0.15.

## Discussion

Primary and incisional hernia repairs still show clinical complications in terms of recurrences, pain and discomfort. Factors like surgical approach, prosthesis characteristics and fixation device and method used may influence surgical outcome.

Laparoscopic ventral/incisional hernia repair has been widely demonstrated to be safe and effective with lower risk of wound infection and shorter hospital stay compared with open repair [2].

The safety of intraperitoneal meshes are supported by over than 20 years of studies on laparoscopic surgery of hernia repair. Several RCTs and meta-analysis of controlled studies published in the last ten years showed that nowadays laparoscopic repair should be considered a safe technique and there is a sufficient follow-up to state that most of the prosthesis provides safe and long lasting strength to the wall. Today, expanded polytetrafluoroethylene (ePTFE), polypropylene (PP), polyethylene terephthalate/polyester (PET), adequately formed or composed with different adhesion barriers, are the main synthetic polymers used [3].

Focusing on PP, A. Baktir [4] studied the formation of collagen after plant of different meshes. They implanted a mesh in five groups of rats divided for type of prothesis: prolene, mersilene, parietex, PTFe and a controlled group. After 30 days, meshes have been removed and analyzed by a histochemical assessment. The study showed that group one (prolene) had a density of collagen statistically superior comparing to other groups (*p* > 0.05). This study supports the use of prolene in the prosthetic repair, because it seems to be the best between materials for this kind of procedures [5–7].

Recently, a new PP composite mesh has become available. It includes two polypropylene layers, a macroporous light mesh, and a thin transparent film (CMC, Clear Composite Mesh, DIPROMED srl San Mauro Torinese, Turin, Italy). The mesh for the parietal side is macroporous, with 88% of porosity and 45 gr/m^2^. It is made of polypropylene monofilament, 120 µm in diameter, to optimize tissue growth. The film for the visceral side is made of non-porous, smooth, transparent polypropylene of thickness 70 µm to prevent the formation of adhesions on the intestinal side. This prosthesis has an ideal design of a composite mesh because it has a visceral side anti-erosive and anti-adhesive function and a ventral macroporous side allowing the growth of fibroblasts. This composite mesh can be colonized by fibroblasts on the side facing the abdominal wall, whereas no cell growth occurs on the side facing the viscera and the temporary inflammation, at early experimental times, is important for healing [8]. In another recent work, based on two series studies in vivo on Wistar rats, CMC prosthesis showed no adhesions to the viscera and no strong foreign body reaction; moreover, its elasticity and anisotropy index were more similar to those of natural tissue [9].

These premises have stimulated us to try this kind of mesh laparoscopically and the results of our experience, although preliminary and limited in number, confirm the good characteristics of this composite mesh.

In our experience, this mesh has been also shown to be able to be easily introduced into the abdominal cavity through 15-mm trocars, and once inside, to be easily handled and positioned in the area of the main abdominal defect.

Fixing of the mesh is another parameter influencing the outcomes of the procedure. Although none of the currently available mesh fixation techniques used in laparoscopy has been found to be superior in preventing hernia recurrence as well as in reducing abdominal wall pain, good results have been reported with non-absorbable spiral tacks [10].

Absorbable devices have been introduced in the market for advertising their property to reduce post-operative pain and intra-abdominal adhesions, and recently few cases series have been reported with satisfactory results [11, 12]. We used absorbable tacks that showed an excellent result in terms of post-operative pain with no incidence of pain and a rate of 0% recurrences.

## Conclusions

Laparoscopic repair should respect tension-free repair concepts, providing a better exposure of the hernia, especially in complex incisional hernias, giving a rapid recovery of the patient, shorter hospital stay and lesser complications because it avoids large detachments like in open technique, besides the other already well known benefits of laparoscopy. Adding to this approach the use of composite mesh in polypropylene fixed with absorbable devices, has proven in our own experience, to be a feasible, safe and above all highly effective with good results although preliminary. Nonetheless, further larger and prospective randomized studies are needed to confirm these results.
